# Methemoglobin Modulation as an Intravascular Contrast Agent for Magnetic Resonance Imaging: Proof of Concept

**DOI:** 10.3389/fvets.2019.00416

**Published:** 2019-11-26

**Authors:** J. Scott McNally, Jared A. Jaffey, Seong-Eun Kim, Matthew D. Alexander, Kate L. Shumway, Leah A. Cohn, Dennis L. Parker, Ronald W. Day

**Affiliations:** ^1^Department of Radiology, Utah Center for Advanced Imaging Research, University of Utah, Salt Lake City, UT, United States; ^2^Department of Medicine and Surgery, Midwestern University College of Veterinary Medicine, Glendale, AZ, United States; ^3^Department of Medicine and Surgery, University of Missouri College of Veterinary Medicine, Columbia, MO, United States; ^4^Department of Pediatrics, University of Utah, Salt Lake City, UT, United States

**Keywords:** animal models, magnetic resonance imaging, magnetic resonance contrast, contrast, hereditary methemoglobinemia, cytochrome b5 reductase deficiency, canine, gadolinium

## Abstract

**Objective:** The aim of this feasibility study was to investigate methemoglobin modulation *in vivo* as a potential magnetic resonance imaging (MRI) gadolinium based contrast agent (GBCA) alternative. Recently, gadolinium tissue deposition was identified and safety concerns were raised after adverse effects were discovered in canines and humans. Because of this, alternative contrast agents are warranted. One potential alternative is methemoglobinemia induction, which can create T1-weighted signal *in vitro*. Canines with hereditary methemoglobinemia represent a unique opportunity to investigate methemoglobin modulation. Our objective was to determine if methemoglobinemia could create high intravascular T1-signal *in vivo* with reversal using methylene blue.

**Methods:** To accomplish this study, a 1.5-year-old male-castrated mixed breed canine with hereditary methemoglobinemia underwent 3T-MRI/MRA with T1-weighted sequences including 3D-T1-weighted Magnetization Prepared Rapid Acquisition Gradient Echo (MPRAGE) and 3D-Time-Of-Flight (TOF). Images were acquired during baseline methemoglobinemia and rescued using intravenous methylene blue (1 mg/kg). Intravascular T1-signal was compared between baseline methemoglobinemia and post-methylene blue. *N* = 10 separate T1-signal measurements were acquired for each vascular structure, normalized to muscle. Significance was determined using paired two-tailed *t*-tests and threshold alpha = 0.05. Fold-change was also calculated using the ratio of T1-signal between methemoglobinemia and post-methylene blue states.

**Results:** At baseline, methemoglobin levels measured 19.5% and decreased to 4.9% after methylene blue. On 3D-T1-weighted MPRAGE, visible signal change was present in internal vertebral venous plexus (IVVP, 1.34 ± 0.09 vs. 0.83 ± 0.05, *p* < 0.001, 1.62 ± 0.06-fold) and external jugular veins (1.54 ± 0.07 vs. 0.87 ± 0.06, *p* < 0.001, 1.78 ± 0.10-fold). There was also significant change in ventral spinal arterial signal (1.21 ± 0.11 vs. 0.79 ± 0.07, *p* < 0.001, 1.54 ± 0.16-fold) but not in carotid arteries (2.12 ± 0.10 vs. 2.16 ± 0.11, *p* = 0.07, 0.98 ± 0.03-fold). On 3D-TOF, visible signal change was in IVVP (1.64 ± 0.14 vs. 1.09 ± 0.11, *p* < 0.001, 1.50 ± 0.11-fold) and there was moderate change in external jugular vein signal (1.51 ± 0.13 vs. 1.19 ± 0.08, *p* < 0.001, 1.27 ± 0.07-fold). There were also small but significant differences in ventral spinal arterial signal (2.00 ± 0.12 vs. 1.78 ± 0.10, *p* = 0.002, 1.13 ± 0.10-fold) but not carotid arteries (2.03 ± 0.17 vs. 1.99 ± 0.17, *p* = 0.15, 1.02 ± 0.04-fold).

**Conclusion:** Methemoglobin modulation produces intravascular contrast on T1-weighted MRI *in vivo*. Additional studies are warranted to optimize methemoglobinemia induction, sequence parameters for maximal tissue contrast, and safety parameters prior to clinical implementation.

## Introduction

Gadolinium based contrast agents (GBCA) utilize T1 and T2 shortening effects to create tissue contrast (1). A large retrospective study in dogs demonstrated few severe adverse effects of GBCA requiring immediate treatment (<1%), though mild to moderate changes in pulse rate, respiratory rate and mean arterial pressure occur on the order of 10 and 20%, respectively (2). Still, more recent studies have demonstrated retention of gadolinium in brains of healthy canines after a single intravenous administration (3). In a previous blood brain barrier disruption model, the addition of gadolinium resulted in a dose-dependent increase in the frequency of delayed seizures (4). These studies raise concern about gadolinium deposition and suggest there may be a dose-related neurotoxicity possible with gadolinium in certain circumstances, but still warrant further study given that the long term effects in normal canines are unknown. Similarly, the use of GBCA in humans is not without potential risk, with known risks of gadolinium in the setting of renal failure and recent research demonstrating gadolinium tissue deposition in those with normal renal function after MRI (5). Because of this, the United States Food and Drug Administration (https://www.fda.gov/Drugs/DrugSafety/ucm589213.htm) and European Medicines Agency (https://www.ema.europa.eu/documents/press-release/emas-final-opinion-confirms-restrictions-use-linear-gadolinium-agents-body-scans_en.pdf) have urged caution and investigation into alternatives.

One potential alternative is methemoglobinemia. Methemoglobin creates T1-hyperintense signal on MRI and can be used to diagnose the subacute stage of intracranial hemorrhage (6), intramural hematoma in dissection (7), and intraplaque hemorrhage (8). Blood methemoglobin levels linearly correlate with T1-weighted signal (*r*^2^ = 0.94, *p* = 0.0015) (9). Methemoglobinemia occurs when ferrous iron (Fe^2+^) in hemoglobin (Hb) is oxidized to ferric iron (Fe^3+^), forming methemoglobin (10, 11). Methemoglobin concentration can be measured directly by blood gas analysis or indirectly with pulse co-oximetry (12). Normal canine and human blood contains ≤ 3% methemoglobin as a result of exposure to endogenous (e.g., by-products of metabolic pathways and reactive oxygen and nitrogen species) and exogenous oxidants (11). Mild methemoglobinemia in humans causes minimal symptoms; however, anxiety, lightheadedness, headache, and tachycardia occur once methemoglobin levels reach 20–30%, and coma, seizures, arrhythmias, acidosis, and death can occur when methemoglobin exceeds 50% (10). While these adverse effects of high levels of methemoglobinemia may be at odds for its use as an alternative contrast agent, low levels of <10% methemoglobinemia can induce measurable changes in MRI contrast (9). If these concentrations can be attained and closely monitored *in vivo*, mild methemoglobinemia induction may serve as an alternative to gadolinium that does not result in heavy metal deposition.

Most methemoglobin reduction (99%) occurs through the cytochrome b_5_ reductase (CYB5R) system also known as the methemoglobin reductase pathway (10). Hereditary defects in the *CYB5R3* gene, which encodes the CYB5R protein, can result in autosomal recessive methemoglobinemia. Two phenotypes of *CYB5R3* mutations are present involving isoforms of the same gene, either restricted to red blood cells (type I, soluble form) or affecting all cells (type II, membrane bound form) (13–15). In erythrocytes, the soluble enzyme reduces methemoglobin (16, 17) whereas the membrane bound form participates in fatty acid, cholesterol and drug metabolism (18–20).

Hereditary methemoglobinemia caused by CYB5R deficiency was first identified in a canine in 1974 and several other reports have since been published in the veterinary literature (21–25). This hereditary condition provides a unique model to investigate the plausibility of modulating methemoglobin levels as an alternative contrast agent. In this model, magnetic resonance imaging (MRI) sequences at baseline represent high methemoglobin levels, which can then be compared to images after normalization of methemoglobin level with administration of methylene blue. Using this canine model, the objective of this proof of concept study was to determine if methemoglobin modulation could create intravascular contrast *in vivo*.

## Materials and Equipment

The study was approved by the Institutional Animal Care and Use Committee at the University of Missouri Veterinary Health Center under protocol #9270 and the owner provided informed consent. A 1.5 year old, male-castrated, mixed breed canine with previously reported hereditary CYB5R deficiency was included in this proof of concept study (23). The canine was otherwise healthy based on physical examination and laboratory investigations (complete blood count, plasma biochemistry, and urinalysis) reviewed by a boarded-certified small animal internist (JAJ). The canine was fasted overnight and presented for participation the next morning. Sedation was accomplished by intramuscular administration of dexmedetomidine (3.3 μg/kg) and nalbuphine (0.3 mg/kg), followed by intravenous administration of propofol for endotracheal intubation. Isoflurane in oxygen was used to maintain adequate anesthesia for imaging. A 20-gauge catheter was placed in a dorsal pedal artery for direct arterial blood pressure measurement throughout anesthesia as well as for whole blood acquisition for serial arterial blood gas analyses. Pulse, respiratory rate, arterial blood pressure, and hemoglobin oxygen saturation were recorded once every 5 min. After baseline MRI sequences were obtained, methylene blue (1 mg/kg) was administered intravenously over 30 min. Four 1 mL arterial blood samples were obtained pre (time 0), and 12, 30, and 45 min after intravenous methylene blue administration. Methemoglobin concentrations were measured using Stat Profile, Prime Plus VET blood gas analyzer (Nova Biomedical, Waltham, MA) within 5 min of acquisition. Methemoglobin was reported as the percentage (MetHb%) of total hemoglobin concentration. Hemoglobin oxygen saturation was measured using standard pulse-oximetry with measurements obtained from the prepuce. After the end of the study at the days' end, the canine was returned to the owner.

### Imaging

3T MRI was performed (Vantage Titan, Canon, Tustin, CA) using a large knee 6-channel array coil. Images were acquired from the brain through the C7 vertebra, pre and 45 min post intravenous methylene blue administration with (1) 3D-Magnetization-Prepared Rapid Acquisition Gradient Echo (MPRAGE) with repetition time (TR) = 6.8 ms, echo time (TE) = 2.7 ms, inversion time (TI) = 800 ms, slice thickness = 1 mm, interslice gap = 0.5 mm, number of acquisitions (NAQ) = 1, acquisition matrix = 208 × 178, flip angle = 9°, field of view (FOV) = 18 × 21 cm and (2) 3D-Time Of Flight (TOF) with TR = 32.0 ms, TE = 3.4 ms, slice thickness = 1 mm, interslice gap = 0.5 mm, NAQ = 1, acquisition matrix = 224 × 224, flip angle = 15^o^, FOV = 23 × 23 cm.

### Image Analysis

Qualitative image analysis was performed on Digital Imaging and Communications in Medicine (DICOM) images and regions of interest (ROI's) were traced using OsiriX. Tissue contrast was measured by calculating signal intensities of veins and arteries of four different structures, with *n* = 10 separate T1-signal measurements for each vascular structure. The following four vascular structures were measured: (1) internal vertebral venous plexus (IVVP), (2) external jugular veins, (3) ventral spinal artery, and (4) carotid arteries. *N* = 10 ROI's were made from the C1 level through C3 on baseline (pre) and copied with equivalent areas for rescue (post methylene blue) images. ROI's were hand drawn fitting each vascular structure but being careful to leave a rim outside of each ROI. Vascular signal was expressed as a multiplicative of muscle signal (M) which was drawn as a replicated circular ROI with equivalent areas on all images. Values were compared during methemoglobinemia (pre) vs. rescue (post methylene blue), with fold-change calculated using the ratio of T1-signal between the two states (methemoglobinemia/methylene blue).

### Statistical Analysis

Stata 14.1 was used for statistical analysis. Significant differences in tissue contrast were determined between methemoglobinemia and post-methylene blue images by using paired two-tailed *t*-tests and threshold alpha = 0.05 from *n* = 10 separate T1-signal measurements acquired per structure. Because *t*-tests are robust to non-normal distributions and heterogeneity of variance, there was no need to test or account for these in our dataset (26–28).

## Results

### Methemoglobinemia and Methylene Blue Treatment

**Figure 1:**
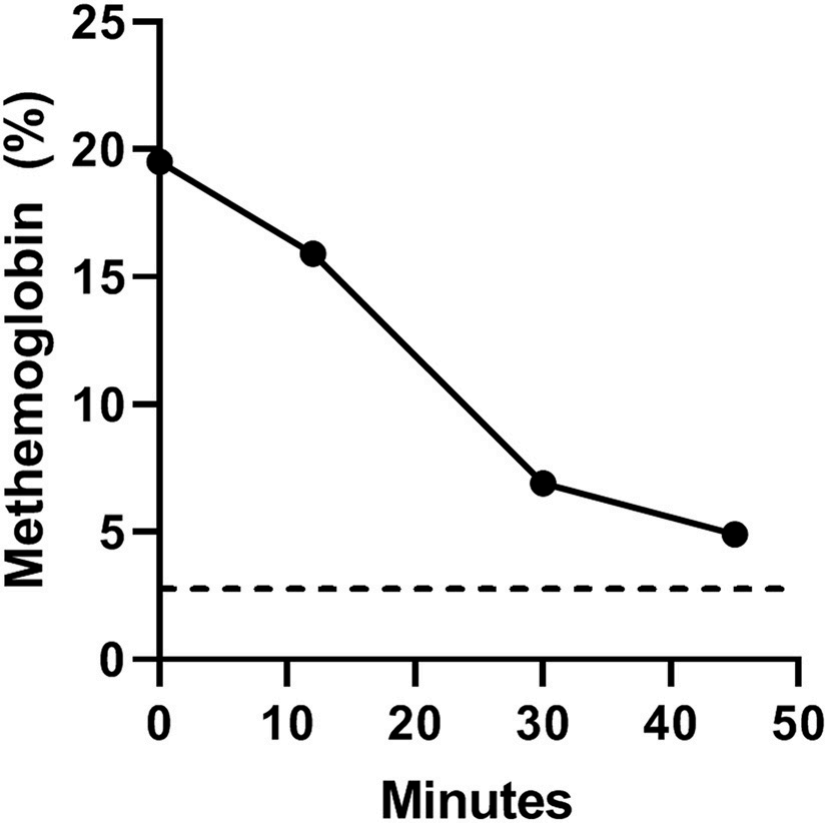
Serial arterial methemoglobin levels (MetHb%) at baseline (time 0) and 12, 30, and 45 min after intravenous methylene blue administration. The black dotted line denotes the upper limit of normal methemoglobin level in canines (<3%).

Methylene blue substantially decreased methemoglobin levels and normalized hemoglobin oxygen saturation. Total hemoglobin content was 16 g/dL (reference range, 12–18 g/dL). Initial hemoglobin oxygen saturation before intravenous methylene blue administration (time 0) was 83% (reference range, >97%) with concomitant inhalant oxygen delivered at 0.5 L/min. Forty-five minutes after intravenous methylene blue administration the hemoglobin oxygen saturation increased to 97%. Likewise, the methemoglobin level gradually decreased from 19.5% (reference range, <3%) at time 0 to 4.9% 45 min after intravenous methylene blue administration (Figure 1).

### 3D-MPRAGE

**Figure 2:**
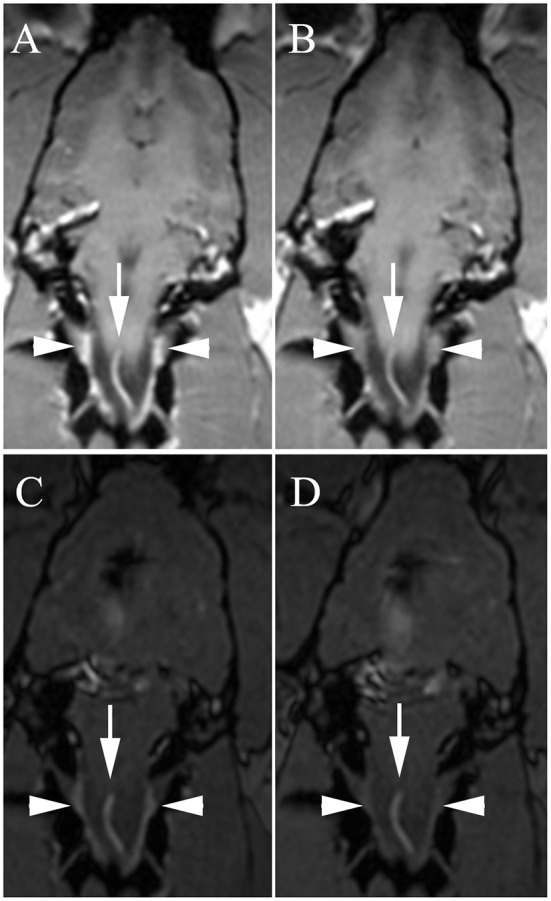
Dorsal 3D-Magnetization Prepared Rapid Acquisition Gradient Echo (MPRAGE) **(A,B)** and dorsal 3D-Time Of Flight (TOF) **(C,D)** with high venous signal at baseline [**A,C**, white arrowheads = internal vertebral venous plexus (IVVP)] and decreased signal after methylene blue **(B,D)**. Arrows, ventral spinal artery.

**Figure 3:**
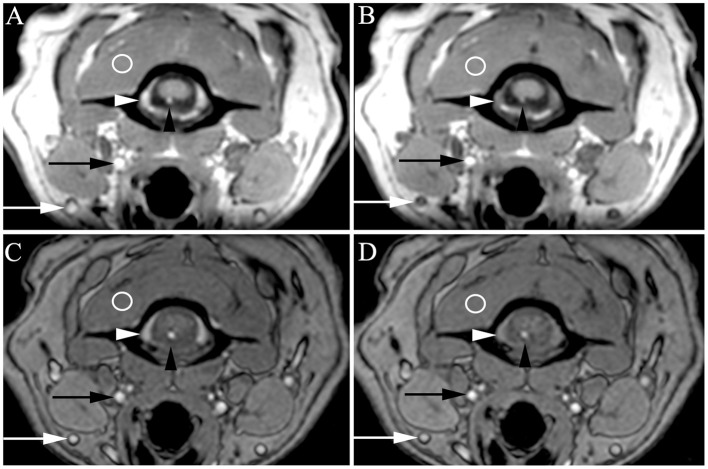
Transverse 3D-Magnetization Prepared Rapid Acquisition Gradient Echo (MPRAGE) **(A,B)** and 3D-Time Of Flight (TOF) **(C,D)** with very high venous signal at baseline [**A,C**, white arrowheads = internal vertebral venous plexus (IVVP), white arrows = external jugular veins] and decreased signal after methylene blue **(B,D)**. Slightly higher arterial signal is also present at baseline (**A,C**, black arrowheads = ventral spinal artery, black arrows = carotid artery) compared to signal after methylene blue **(B,D)**, though somewhat masked by background arterial flow related enhancement intrinsic to these sequences. Regions of interest in veins and arteries were normalized to muscle (circles) and compared between baseline and methylene blue. On MPRAGE, these representative images demonstrated increased signal compared to muscle as follows: ventral spinal artery pre vs. post 1.25 X muscle (1.25 M) vs. 0.76 M, IVVP pre vs. post = 1.39 vs. 0.81 M. On TOF, representative images demonstrated increased signal compared to muscle as follows: ventral spinal artery pre vs. post 1.93 vs. 1.75 M, IVVP pre vs. post = 1.60 vs. 1.10 M.

**Figure 4:**
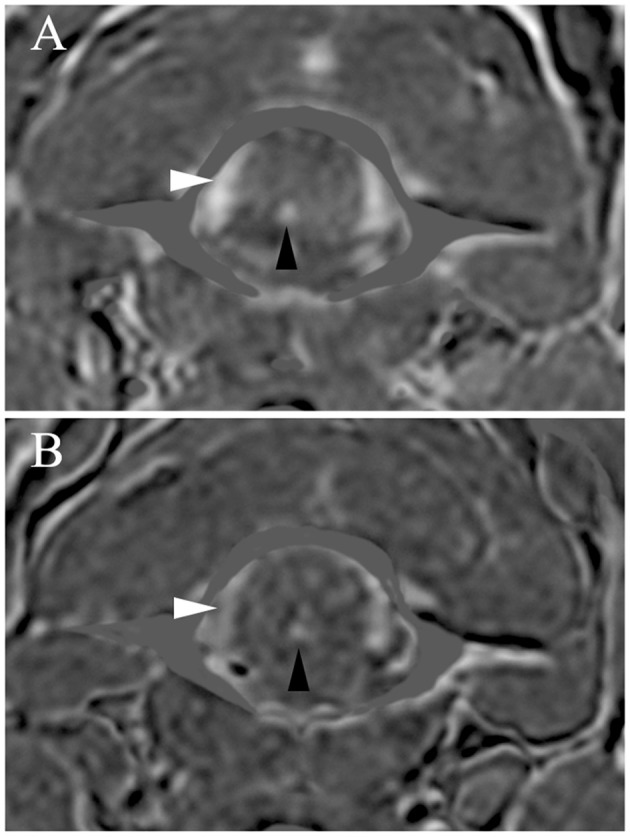
Transverse subtraction data from 3D-Magnetization Prepared Rapid Acquisition Gradient Echo (MPRAGE) **(A)** and 3D-Time Of Flight (TOF) images **(B)**. High relative signal is present on the MPRAGE subtraction images **(A)** in the veins (IVVP, white arrowheads) and arteries (ventral spinal artery, black arrowheads). MPRAGE fold increases in signal (ratio of signal during methemoglobinemia/methylene blue) were for the ventral spinal artery = 1.57-fold and for the IVVP = 1.66-fold. There was also high relative signal on TOF subtraction images **(B)** in the veins (IVVP, white arrowheads) and arteries (ventral spinal artery, black arrowheads). TOF fold increases in signal (ratio of signal during methemoglobinemia/methylene blue) were for the ventral spinal artery = 1.25-fold and for the IVVP = 1.54-fold.

**Figure 5:**
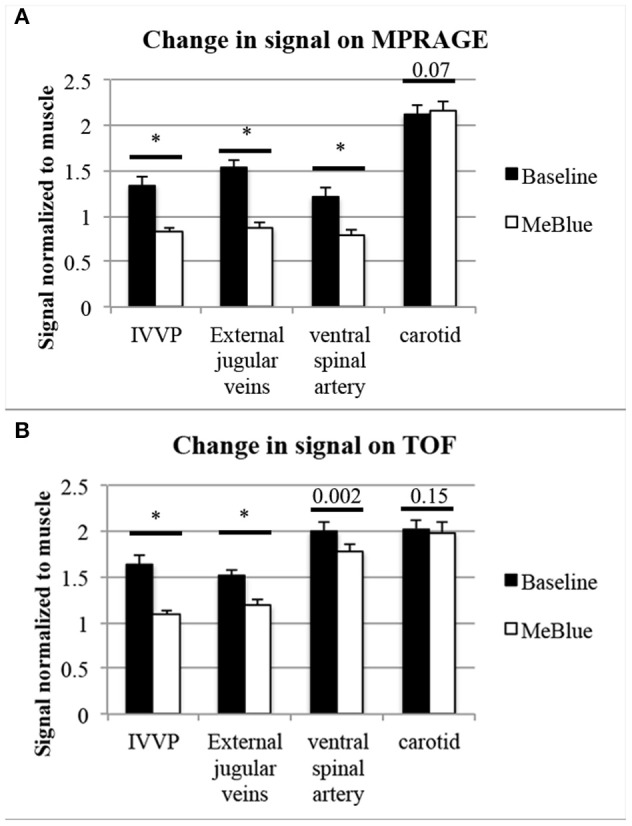
Pooled data showing intravascular T1-signal at baseline compared to post-methylene blue (both normalized to muscle signal). *N* = 10 separate T1-signal measurements were acquired per structure. *P*-values from two-tailed *t*-tests were placed above comparisons between baseline methemoglobinemia (black bars) and post-methylene blue states (white bars), with asterisks* indicating *p* < 0.001. On 3D-MPRAGE **(A)**, there were significant changes between baseline and methylene blue in IVVP and external jugular veins and slightly less so in the ventral spinal artery, but the difference in carotid artery signal was minimal and did not meet our significance threshold of *p* < 0.05. On 3D-TOF **(B)**, there were moderate but significant changes in IVVP signal, slightly less though significant changes in external jugular vein signal, and mild but significant changes in the ventral spinal artery signal, but no significant change in carotid artery signal.

On 3D-MPRAGE, the most visible signal change was in the internal vertebral venous plexus (IVVP) and external jugular veins, and less so involving the ventral spinal artery but not in carotid arteries (Table 1 and Figures 2–5). IVVP signal change was 1.62 ± 0.06-fold (*p* < 0.001), external jugular vein signal was 1.78 ± 0.10-fold (*p* < 0.001), ventral spinal artery signal was 1.54 ± 0.16-fold (*p* < 0.001).

**Table 1:** Change in signal on 3D-MPRAGE of internal vertebral venous plexus (IVVP), external jugular veins, ventral spinal artery, and carotid arteries comparing pre (methemoglobinemia) to post-methylene blue.

**3D-MPRAGE**	**Baseline**	**Post me blue**	**Fold change**	***p*-value**
	**(X muscle)**	**(X muscle)**		
IVVP	1.34 ± 0.09	0.83 ± 0.05	1.62 ± 0.06	<0.001
External jugular veins	1.54 ± 0.07	0.87 ± 0.06	1.78 ± 0.10	<0.001
Ventral spinal artery	1.21 ± 0.11	0.79 ± 0.07	1.54 ± 0.16	<0.001
Carotid arteries	2.12 ± 0.10	2.16 ± 0.11	0.98 ± 0.03	0.07

*N = 10 separate T1-signal measurements were acquired for each structure*.

### 3D-TOF

On 3D-TOF, the most visible signal change was in IVVP. There was also moderate change in external jugular vein signal. There was a smaller but significant difference in ventral spinal arterial signal but carotid artery signal was not different (Table 2 and Figures 2–5). IVVP signal change was 1.50 ± 0.11-fold (*p* < 0.001), external jugular vein signal change was 1.27 ± 0.07-fold (*p* < 0.001), and ventral spinal arterial signal change was 1.13 ± 0.10-fold (*p* = 0.002).

**Table 2:** Change in signal on 3D-TOF of internal vertebral venous plexus (IVVP), external jugular veins, ventral spinal artery, and carotid arteries comparing pre (methemoglobinemia) to post-methylene blue.

**3D-TOF**	**Baseline**	**Post me blue**	**Fold change**	***p*-value**
	**(X muscle)**	**(X muscle)**		
IVVP	1.64 ± 0.14	1.09 ± 0.11	1.50 ± 0.11	<0.001
External jugular veins	1.51 ± 0.13	1.19 ± 0.08	1.27 ± 0.07	<0.001
Ventral spinal artery	2.00 ± 0.12	1.78 ± 0.10	1.13 ± 0.10	0.002
Carotid arteries	2.03 ± 0.17	1.99 ± 0.17	1.02 ± 0.04	0.15

*N = 10 separate T1-signal measurements were acquired for each structure*.

## Discussion

This feasibility study created T1-weighted tissue contrast on MRI using *in vivo* methemoglobin modulation. This technique may have potential as an alternative intravascular contrast agent in both humans and canines. This is supported by the significant increase in contrast signal on standard T1-weighted sequences obtained at time 0 (MetHb% of 19.5%) compared to those obtained 45 min after intravenous methylene blue administration (MetHb% of 4.9%).

While MR contrast agents in veterinary patients are primarily used to evaluate brain, spine, and other structures for abnormal enhancement as opposed to vascular pathology, in some cases MR vascular imaging may be useful. Future applications of MR vascular imaging may include preoperative planning or characterization of vascular pathology as a non-invasive alternative to catheter angiography. While vascular imaging is used extensively in humans for diagnostic use, the true incidence of cerebrovascular disease in animals is unknown (29). Veterinary vascular imaging may be useful to assess this incidence in a non-invasive manner, and may be useful clinically to assess feeding vessels in tumors or diagnose vascular pathology including hemorrhages (30, 31), thrombosis (31), congenital vascular malformations (32), intravascular cerebral lymphoma (33), aneurysms, and post traumatic arteriovenous fistulas (34), all being potential clinical indications in veterinary patients. MRA may also play a future role in the diagnosis of acute ischemic stroke as this has been increasingly recognized in canines (35, 36). Case reports have shown that MRA can non-invasively study normal canine vascular anatomy as well as feeding vessels in veterinary patients with meningiomas or arteriovenous fistulas (37). MRA may also add value in pre-operative tumor typing between canine meningioma and intracranial histiocytic sarcoma (38).

One limitation of this study is the focus on a single canine with hereditary methemoglobinemia. While the current canine model took advantage of baseline methemoglobinemia related to hereditary CYB5R deficiency and used methylene blue for rescue, clinical applications would require methemoglobinemia induction. An essential next step is to induce and modulate methemoglobinemia levels in a healthy animal model to determine safety and efficacy prior to human studies. The United States Food and Drug Association has approved two agents that can induce methemoglobinemia in humans: (1) sodium nitrite is approved to treat cyanide toxicity and (2) inhaled nitric oxide is approved to treat newborns with pulmonary hypertension and acute respiratory failure. A recent experimental study in canines demonstrated that the use of intravenous sodium nitrite was safe and well-tolerated in canines when induced methemoglobin levels were ≤ 40% (39).

Controlled induction of methemoglobinemia as a contrast agent has advantages and disadvantages. One benefit of methemoglobinemia is that it remains within erythrocytes and is a true intravascular contrast agent that can be used for blood pool imaging. This could also be viewed as a disadvantage, and parenchymal enhancement may not be present without capillary leakage or active extravasation. Future studies could envision induction of methemoglobinemia in animal models of blood brain barrier injury to test this possibility. Methemoglobinemia can be monitored non-invasively using pulse co-oximetry and titrated to avoid toxicity. Standard pulse oximetry in patients with methemoglobinemia is not useful because methemoglobin absorbs both infrared and red light equally, which interferes with the measured percentage of oxyhemoglobin and deoxyhemoglobin as was seen at time 0 (hemoglobin oxygen saturation of 83%) (10). In contrast to standard pulse oximetry, a co-oximeter measures light absorbance at four different wavelengths, which allows for characterization of methemoglobin with a peak absorbance of light at 630 nm (10). A pulse co-oximeter was not available for this study, which necessitated the measurement of arterial methemoglobin levels. Another benefit of utilizing controlled induction of methemoglobinemia as a contrast agent is that it can rapidly and safely be reversed with intravenous administration of methylene blue if concern for toxicity develops. As an alternative, ascorbic acid has also been used to reverse methemoglobinemia in case reports (40), though in canines this was used in combination with N-acetylcysteine for concomitant acetaminophen toxicity (41). In the absence of toxicity, there is no need to administer antidotal therapy like methylene blue because methemoglobin reductase, the primary endogenous redox pathway quickly reduces methemoglobin to hemoglobin. In fact, the half-life of methemoglobin in a patient with normal methemoglobin reductase function is 55 min (42).

While methemoglobinemia can transiently decrease oxygen carrying capacity, supplemental oxygen might partially obviate its impact sufficiently to limit potential toxicity. Methemoglobin levels below 30% in a healthy person produce minimal symptoms (10). However, methemoglobin levels of 30–50% can result in cardiovascular and central nervous system derangements (e.g., weakness, tachycardia, tachypnea, and mild dyspnea) and levels ≥50% can result in coma, seizures, and arrhythmias (10). Methemoglobin and clinical correlation studies have not been performed in canines but are postulated to be similar to humans. Importantly, these clinical predictions are based on the assumption that a human patient has a total hemoglobin concentration of 15 g/dL and lacks comorbid conditions that could decrease arterial oxygen tension or perfusion. It is clear that future studies investigating methemoglobinemia as a contrast agent in canines and humans should focus on achieving the lowest possible level of methemoglobinemia to adequately increase the T1-weighted signal intensity of blood. Further, methemoglobin modulation might be inappropriate, or may need to be used with added caution, in vulnerable canines and humans with decreased oxygen delivery. Anemia or cardiopulmonary disease may increase risk of adverse effects associated with a given methemoglobin level (10).

In conclusion, this feasibility study shows that methemoglobinemia could serve as a potential alternative MRI contrast agent in canines and humans. This technique may apply to a subset of patients and indications, particularly those that require intravascular contrast. Additional studies are necessary prior to any recommendations on this technique. Future studies could be performed on the various methods of methemoglobinemia induction, sequences used for maximal tissue contrast, and safety parameters prior to clinical implementation.

## Data Availability Statement

The datasets generated for this study are available on request to the corresponding author.

## Ethics Statement

The animal study was reviewed and approved by Institutional Animal Care and Use Committee at the University of Missouri Veterinary Health Center under protocol #9270. Written informed consent was obtained from the owners for the participation of their animals in this study.

## Author Contributions

JM, S-EK, MA, DP, and RD contributed conception and design of the study. JJ, KS, and LC performed the MR imaging. JM and S-EK performed the statistical analysis. JM wrote the first draft of the manuscript. JJ, KS, and RD wrote sections of the manuscript. All authors contributed to manuscript revision, read and approved the submitted version.

### Conflict of Interest

The authors declare that the research was conducted in the absence of any commercial or financial relationships that could be construed as a potential conflict of interest.
